# Trends in Clinical, Demographic, and Biochemical Characteristics of Patients With Acute Myocardial Infarction From 2003 to 2008: A Report From the American Heart Association Get With The Guidelines Coronary Artery Disease Program

**DOI:** 10.1161/JAHA.112.001206

**Published:** 2012-08-24

**Authors:** Nathan M. Boyer, Warren K. Laskey, Margueritte Cox, Adrian F. Hernandez, Eric D. Peterson, Deepak L. Bhatt, Christopher P. Cannon, Gregg C. Fonarow

**Affiliations:** 1 University of New Mexico, Albuquerque, NM (N.M.B., W.K.L.); 2 Duke Clinical Research Institute, Durham, NC (M.C., A.F.H., E.D.P.); 3 VA Boston Healthcare System, Brigham and Women's Hospital and Harvard Medical School, Boston, MA (D.L.B.); 4 Brigham and Women's Hospital and Harvard Medical School, Boston, MA (C.P.C.); 5 University of California, Los Angeles, CA (G.C.F.)

**Keywords:** coronary disease, epidemiology, myocardial infarction, population, risk factors

## Abstract

**Background:**

An analysis of the changes in the clinical and demographic characteristics of patients with acute myocardial infarction could identify successes and failures of risk factor identification and treatment of patients at increased risk for cardiovascular events.

**Methods and Results:**

We reviewed data collected from 138 122 patients with acute myocardial infarction admitted from 2003 to 2008 to hospitals participating in the American Heart Association Get With The Guidelines Coronary Artery Disease program. Clinical, demographic, and laboratory characteristics were analyzed for each year stratified on the electrocardiogram at presentation. Patients with non–ST-segment–elevation myocardial infarction were older, more likely to be women, and more likely to have hypertension, diabetes mellitus, and a history of past cardiovascular disease than were patients with ST-elevation myocardial infarction. In the overall patient sample, significant trends were observed of an increase over time in the proportions of non–ST-segment–elevation myocardial infarction, patient age of 45 to 65 years, obesity, and female sex. The prevalence of diabetes mellitus decreased over time, whereas the prevalences of hypertension and smoking were substantial and unchanging. The prevalence of “low” high-density lipoprotein increased over time, whereas that of “high” low-density lipoprotein decreased. Stratum-specific univariate analysis revealed quantitative and qualitative differences between strata in time trends for numerous demographic, clinical, and biochemical measures. On multivariable analysis, there was concordance between strata with regard to the increase in prevalence of patients 45 to 65 years of age, obesity, and “low” high-density lipoprotein and the decrease in prevalence of “high” low-density lipoprotein. However, changes in trends in age distribution, sex ratio, and prevalence of smokers and the magnitude of change in diabetes mellitus prevalence differed between strata.

**Conclusions:**

There were notable differences in risk factors and patient characteristics among patients with ST-elevation myocardial infarction and those with non–ST-segment–elevation myocardial infarction. The increasing prevalence of dysmetabolic markers in a growing proportion of patients with acute myocardial infarction suggests further opportunities for risk factor modification. **(*J Am Heart Assoc*. 2012;1:e001206 doi: 10.1161/JAHA.112.001206.)**

## Introduction

Description of the behavioral, environmental, and genetic factors in patients with acute myocardial infarction (AMI) underscores our current understanding of the causal relationship between patient- and population-specific exposures, or risk factors, and clinical outcomes.^1–4^ Patients with AMI represent a distinct, highly select subgroup of the general population. Changes in the extent and distribution of specific clinical, demographic, and biochemical factors over time in patients with AMI provide insight into the overall burden of disease in individuals at the highest risk for AMI. The latter is of relevance from demographic and public health perspectives, given the increasing number of individuals in the general population at risk for AMI^5^ and the increasing number of survivors of AMI.^6^ Finally, such studies, by revealing an increased or unchanging presence of specific risk factors, could suggest additional or missed opportunities for preventive strategies.^7–8^

In the present analysis from the American Heart Association (AHA) Get With The Guidelines Coronary Artery Disease (GWTG-CAD) program, we report the prevalences of clinical, demographic, and biochemical factors in patients presenting with AMI and the changes in those prevalences from 2003 to 2008.

## Methods

### The AHA GWTG-CAD Program

The mission, scope, and purpose of the AHA GWTG-CAD program have been described previously.^9–10^ Because GWTG-CAD is a quality-improvement program, hospitals are encouraged to consecutively enroll all eligible patients. The GWTG-CAD population includes all patients admitted to the hospital who were subsequently discharged with a diagnosis of AMI, unstable angina, chronic stable angina, or ischemic heart disease (*International Classification of Diseases, Ninth Revision, Clinical Modification* [*ICD-9-CM*] codes 410–414). Each participating site is responsible for its own data collection and uploading. Data quality is monitored in a Web-based system, and reports are provided to the site to ensure completeness and accuracy of the submitted data. Data collected include patient demographics, medical history, symptoms on arrival, results of laboratory testing, in-hospital treatment and events, discharge treatment and counseling, and patient disposition. The de-identification of patients occurs at this level.

All participating institutions were required to comply with local regulatory and privacy guidelines and to submit the GWTG-CAD protocol for review and approval by their institutional review boards. Because data were used primarily at the local site for quality improvement, sites were granted a waiver of informed consent under the common rule. The Duke Clinical Research Institute (Durham, NC) serves as the data analysis center and has institutional review board approval to analyze the aggregate de-identified data for research purposes.

### Patient Population

The GWTG-CAD program began in 2000, and the length of participation of each hospital depended on the time it entered the program. Baseline data included the first 30 admissions for each participating site and served as the entry point into the study. Subsequently, participation time was calculated in calendar quarters. Quarters with <1000 admissions were excluded to obtain reliable estimates of trends over time; this necessitated exclusion of data obtained in all 4 quarters of 2000 and 2001. Therefore, all GWTG-CAD–participating hospitals enrolled from January 1, 2002, to April 2010 were eligible for analysis.

The patient sample for this study was derived from the population of patients with a first-listed diagnosis and supporting *ICD-9-CM* code for coronary heart disease who were admitted to hospitals participating in the AHA GWTG-CAD program. Data from January 2002 through April 2010 were reviewed. Over this interval, 282 585 patients had an *ICD-9-CM*–consistent diagnosis of AMI (*ICD-9-410*). Excluded were records created before 2003 (n=23 024), records created after 2008 because of administrative changes in the GWTG program (n=16 396), patients with heart failure with CAD (n=36 574), patients without an AMI (n=66 940), and patients with an unspecified AMI (n=1529). The final study sample consisted of 138 122 patients (from 398 sites) admitted from January 1, 2003, to December 31, 2008. Patients subsequently were categorized by the electrocardiogram pattern on admission: specifically, those with ST-segment–elevation myocardial infarction (STEMI; n=44 172) or a new or presumably new left bundle-branch block pattern and those without ST-segment elevation (NSTEMI; n=92 950).

### Data and Statistical Analysis

Data are presented as means ± standard deviations or medians and interquartile ranges (IQRs) for continuous variables and as percentages for categorical variables for the overall data set and separately for the STEMI and NSTEMI strata. Univariate associations between categorical variables and year of observation (ordered variable) were tested with χ^2^ statistics (for >3 levels per categorical variable) and Wilcoxon rank-based statistics (for 2 levels per category). The overall effect of linear yearly trend for each variable of interest was tested with the Cochran-Mantel-Haenszel method. *P* values for continuous data are based on χ^2^ 1-degree-of-freedom rank correlation statistics. Stratum-specific multivariable logistic regression was performed to assess the association of time and the following dichotomous risk factors for AMI: age, sex, history of hypertension, history of prior myocardial infarction, history of treated diabetes mellitus, history of current or recent smoking, obesity (body mass index [BMI] >30 kg/m^2^), dyslipidemia (low-density lipoprotein [LDL] >100 mg/dL; high-density lipoprotein [HDL] <40 mg/dL for men, <50 mg/dL for women; triglycerides >150 mg/dL). Because patients admitted to the same hospital can have similar characteristics, the generalized estimating equations method with an exchangeable working correlation structure was used to adjust for within-hospital clustering.^11^ The generalized estimating equations method is only one analytical strategy to handle correlations within the same hospital. The generalized estimating equations method does not control for potential confounding effects due to the different types of hospitals to which patients are admitted. Therefore, hospital-level variables are included in the regression. Potential confounding variables were included in each fitted model for each designated risk characteristic outcome. These variables included the following baseline characteristics: age, sex, race (white, black/African American, Hispanic origin, other), BMI, insurance status, atrial fibrillation, chronic obstructive pulmonary disease, diabetes mellitus, hyperlipidemia, hypertension, peripheral vascular disease, prior myocardial infarction, heart failure, dialysis, renal insufficiency, current/recent smoking, United States Census–defined geographic region, number of beds, teaching status, and cardiac surgery on site. Age, BMI, and number of beds were entered as continuous variables, and missing values were imputed from the median. Age had no missing data. Patients whose sex was missing from the data were excluded from modeling because of concerns about data quality for other variables. Insurance status was categorized as Medicare, Medicaid, other insurance, and no insurance. Less than 9% of insurance data were missing. Patients ≥65 years of age were imputed to Medicare. All other patients were imputed to other insurance, because this category is more likely (no insurance or Medicaid is more likely to be recorded by a data entry specialist). Medical history panel variables were missing in 5.9% of patients; missing values were imputed to “no” because of hypothesized omissions. Race was missing in 2.3% of patients; missing values were imputed to “white.” BMI was imputed to the sex-specific median for 10.9% of patients (10.5% after exclusion of patients with sex missing).

A variable was not included in the model as an independent variable when that variable was the dependent variable. From these models, unadjusted and adjusted odds ratios (ORs) for the change in prevalence of each analyzed risk factor per quarter–calendar-year increment were estimated, and results were reported as the cumulative OR for the 6 years of the study by exponentiating the OR per 1-year change to the power of 6. Because there was evidence for statistical interaction—that is, *P*<0.05—in several of the models (male sex, diabetes mellitus, hypertension) when the interaction term (time×STEMI/NSTEMI) was added to the above list of confounders, results are reported separately for each stratum.

### Sensitivity Analysis

Because sites both “dropped in” and “dropped out” over the time interval of the study, a sensitivity analysis was performed on only those sites that contributed at least 1 patient in 2003 or 2004 and at least 1 patient in each of the following years: 2005, 2006, 2007, and 2008. For this “core” data set analysis, there were 73 715 patients from 78 unique sites.

All analyses were performed in SAS version 9.2 (SAS Institute, Cary, NC). All significance tests were 2 sided, and *P* values <0.05 were considered statistically significant. Given the number of comparisons performed in this study, the lack of adjustment for such multiple comparisons, and the large overall sample size, a more conservative definition of statistical significance is suggested when *P*<0.001.

## Results

### Demographic Characteristics of Overall AMI Patient Population Over Time: Univariate Analysis

**Figure 1.**
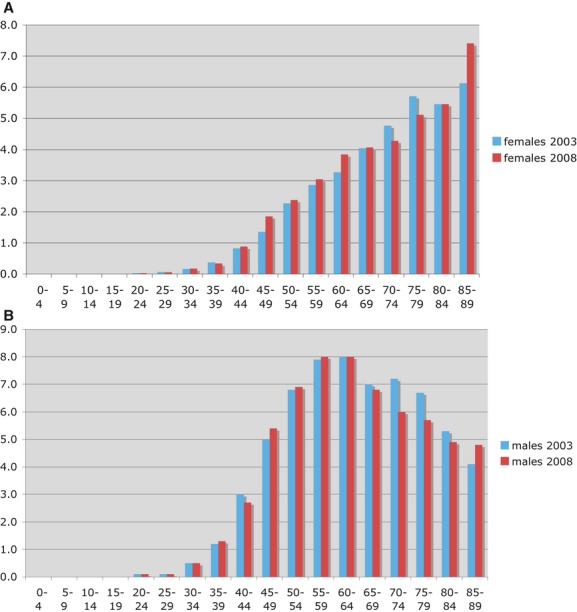
A, Distribution of women with AMI in 2003 (blue) and 2008 (red) in the AHA GWTG-CAD sample. Women >70 years of age comprised the highest proportion of female patients with AMI. There was an increase in the percentage of patients (*x*-axis) in the 45- to 65-year age group between 2003 and 2008. These age cohorts correspond to a significant portion of the “Baby Boom” generation born between 1946 and 1964. There is also an increase in the proportion of older women with AMI (>85 years) from 2003 to 2008. B, Distribution of men with AMI in 2003 (blue) and 2008 (red) in the AHA GWTG-CAD sample. Men between 55 and 65 years of age comprised the highest proportion of male patients with AMI. There was an increase in the percentage of patients (*x*-axis) in the 45- to 65-year age group between 2003 and 2008. These age cohorts correspond to a significant portion of the “Baby Boom” generation born between 1946 and 1964. There is also an increase in the proportion of older men with AMI (>85 years) from 2003 to 2008.

As seen in Table 1, the change in age distribution over time for the total sample was of borderline statistical significance (2003 median/IQR, 67/22 years; 2008 median/IQR, 66/22 years; *P*=0.0535). However, there was a significant increase over time in the relative proportion of patients between the ages of 45 and 65 years (*P*<0.0001), and the relative proportion of patients ≥65 years of age decreased (*P*<0.0001). There was a significant change in the sex ratio (males per 100 females) from 2003 to 2008, with an increasing proportion of females in later years (Figure 1A and 1B).

**Table 1.** Trends in Demographic, Medical Historical, and Laboratory Characteristics in the Overall AMI Patient Sample

Variables	Level	Overall (n=138 122)		2003 (n=19 504)		2004 (n=22 177)		2005 (n=27 689)		2006 (n=22 921)		2007 (n=23 086)		2008 (n=22 745)		*P*
Diagnosis	NSTEMI	92 950	67.30	12 798	65.62	14 691	66.24	19 051	68.80	15 281	66.67	15 438	66.87	15 691	68.99	<0.0001
Demographics																
Age	Median	138 122	67.00	19 504	67.00	22 177	67.00	27 689	67.00	22 921	66.00	23 086	66.00	22 745	66.00	0.0535
	25th		55.00		56.00		56.00		56.00		55.00		55.00		56.00	
	75th		78.00		78.00		78.00		78.00		78.00		78.00		78.00	
	Mean		66.43		66.66		66.45		66.52		66.08		66.29		66.60	
	SD		14.43		14.10		14.38		14.51		14.42		14.53		14.53	
	Minimum		18.00		19.00		18.00		18.00		19.00		18.00		19.00	
	Maximum		107.00		104.00		106.00		104.00		105.00		106.00		107.00	
Age ≤45 y	Yes	10 645	7.71	1446	7.41	1717	7.74	2196	7.93	1812	7.91	1808	7.83	1666	7.32	0.7521
Age ≥65 y	Yes	75 473	54.64	10 980	56.30	12 254	55.26	15 260	55.11	12 238	53.39	12 362	53.55	12 379	54.43	<0.0001
Age >45, <65 y	Yes	52 004	83.01	7078	83.04	8206	82.70	10 233	82.33	8871	83.04	8916	83.14	8700	83.93	0.0241
Sex	Female	51 496	37.28	7251	37.18	8365	37.72	10 389	37.52	8389	36.60	8586	37.19	8516	37.44	0.0024
Race	Other	5424	3.93	619	3.17	681	3.07	1133	4.09	959	4.18	995	4.31	1037	4.56	<0.0001
	Hispanic	10 051	7.28	1549	7.94	2023	9.12	2169	7.83	1579	6.89	1321	5.72	1410	6.20	
	Black or African American	10 181	7.37	1390	7.13	1563	7.05	1892	6.83	1778	7.76	1692	7.33	1866	8.20	
	White	10 3250	74.75	14 667	75.20	16 092	72.56	20 579	74.32	16 885	73.67	17 920	77.62	17 107	75.21	
	Missing	9216	6.67	1279	6.56	1818	8.20	1916	6.92	1720	7.50	1158	5.02	1325	5.83	
Non-Hispanic white	Yes	10 3250	74.75	14 667	75.20	16 092	72.56	20 579	74.32	16 885	73.67	17 920	77.62	17 107	75.21	<0.0001
Hispanic	Yes	10 051	7.28	1549	7.94	2023	9.12	2169	7.83	1579	6.89	1321	5.72	1410	6.20	<0.0001
Insurance	No insurance/not documented/UTD	12 268	8.88	1395	7.15	2361	10.65	2958	10.68	2193	9.57	1686	7.30	1675	7.36	<0.0001
	Medicare	42 009	30.41	6492	33.29	7157	32.27	8886	32.09	6510	28.40	6377	27.62	6587	28.96	
	Medicaid	9268	6.71	1096	5.62	1615	7.28	1939	7.00	1648	7.19	1531	6.63	1439	6.33	
	Other	63 227	45.78	7719	39.58	10 517	47.42	13 629	49.22	10 873	47.44	10 715	46.41	9774	42.97	
	Missing	11 350	8.22	2802	14.37	527	2.38	277	1.00	1697	7.40	2777	12.03	3270	14.38	
Medical history																
None	Yes	12 309	9.47	1387	7.73	1730	8.22	2337	8.83	2236	10.22	2481	11.38	2138	10.28	<0.0001
Chronic or recurrent atrial fibrillation	Yes	10 018	7.71	1406	7.83	1743	8.28	2281	8.62	1589	7.26	1404	6.44	1595	7.67	<0.0001
Atrial flutter	Yes	417	0.32	0	0.00	0	0.00	3	0.01	108	0.49	153	0.70	153	0.74	<0.0001
COPD or asthma	Yes	18 429	14.18	2717	15.14	2997	14.23	3558	13.45	2923	13.36	3059	14.03	3175	15.26	0.7310
Diabetes mellitus	Yes	41 623	32.03	5966	33.24	7043	33.44	8603	32.51	6747	30.85	6680	30.63	6584	31.65	<0.0001
Hyperlipidemia	Yes	60 750	46.75	5868	32.69	9680	45.97	13 187	49.84	10 922	49.93	10 614	48.67	10 479	50.37	<0.0001
Hypertension	Yes	87 194	67.10	12 139	67.63	14 213	67.49	17 897	67.64	14 378	65.73	14 317	65.65	14 250	68.50	0.1931
Peripheral vascular disease	Yes	11 276	8.68	1734	9.66	1876	8.91	2253	8.51	1854	8.48	1758	8.06	1801	8.66	<0.0001
Prior MI/CAD	Yes	38 429	29.57	4192	23.35	4481	21.28	5262	19.89	7174	32.80	8681	39.81	8639	41.53	<0.0001
CVA/TIA	Yes	11 141	8.57	1583	8.82	1707	8.11	1955	7.39	1748	7.99	2105	9.65	2043	9.82	<0.0001
Heart failure	Yes	19 580	15.07	2725	15.18	3576	16.98	4251	16.07	3153	14.42	2906	13.33	2969	14.27	<0.0001
Anemia	Yes	2897	2.23	0	0.00	0	0.00	1	0.00	668	3.05	1063	4.87	1165	5.60	<0.0001
Renal insufficiency	Yes	12 445	9.58	2056	11.45	2358	11.20	2653	10.03	1925	8.80	1769	8.11	1684	8.09	<0.0001
Depression	Yes	3436	2.64	1	0.01	0	0.00	6	0.02	678	3.10	1336	6.13	1415	6.80	<0.0001
Prior PCI	Yes	2695	2.07	0	0.00	0	0.00	0	0.00	1	0.00	50	0.23	2644	12.71	<0.0001
Prior CABG	Yes	1980	1.52	0	0.00	0	0.00	0	0.00	0	0.00	43	0.20	1937	9.31	<0.0001
Medical history panel missing	Yes	8168	5.91	1554	7.97	1118	5.04	1228	4.43	1048	4.57	1278	5.54	1942	8.54	<0.0001
Smoking	Yes	43 010	31.14	5834	29.91	6924	31.22	8508	30.73	7315	31.91	7261	31.45	7168	31.51	0.7434
Laboratories																
BMI	Median	123 127	27.59	18 023	27.40	20 688	27.41	25 670	27.49	19 943	27.62	19 725	27.83	19 078	27.89	<0.0001
	25th		24.22		24.21		24.12		24.16		24.22		24.34		24.38	
	75th		31.74		31.37		31.37		31.59		31.95		32.17		32.09	
	Mean		28.54		28.29		28.27		28.43		28.62		28.83		28.79	
	SD		6.62		6.43		6.44		6.56		6.68		6.88		6.71	
	Minimum		13.02		13.05		13.03		13.02		13.07		13.04		13.04	
	Maximum		99.27		99.27		96.95		98.41		88.71		97.00		96.88	
BMI ≥30 kg/m^2^	Yes	41 860	30.31	5776	29.61	6636	29.92	8530	30.81	6943	30.29	7095	30.73	6880	30.25	<0.0001
Total cholesterol, mg/dL	Median	94 094	167.00	12 791	173.00	14 868	169.00	18 749	167.00	15 754	165.00	16 169	164.00	15 763	163.00	<0.0001
	25^th^		138.00		145.00		141.00		139.00		136.00		135.00		134.00	
	75^th^		199.00		205.00		201.00		199.00		198.00		195.00		196.00	
	Mean		171.06		177.67		173.84		171.29		169.61		167.93		167.45	
	SD		47.88		47.53		47.36		47.20		48.30		48.35		47.86	
	Minimum		10.00		15.00		16.00		10.00		11.00		21.00		18.00	
	Maximum		827.00		776.00		720.00		592.00		667.00		827.00		642.00	
Total cholesterol >200 mg/dL	Yes	22 551	16.33	3604	18.48	3769	17.00	4504	16.27	3661	15.97	3548	15.37	3465	15.23	<0.0001
HDL, mg/dL	Median	92 601	37.00	12 430	39.00	14 574	38.00	18 490	36.00	15 538	36.00	15 990	36.00	15 579	36.00	<0.0001
	25^th^		30.00		32.00		31.00		29.00		30.00		30.00		30.00	
	75^th^		45.00		47.00		46.00		45.00		45.00		45.00		45.00	
	Mean		38.77		40.57		39.50		37.93		38.29		38.49		38.40	
	SD		12.89		12.73		13.19		13.57		12.87		12.24		12.42	
	Minimum		0.00		0.00		0.00		0.00		0.00		0.00		2.00	
	Maximum		100.00		100.00		100.00		100.00		100.00		100.00		100.00	
HDL <40 mg/dL	Yes	54 836	39.70	6562	33.64	8200	36.98	11 265	40.68	9510	41.49	9714	42.08	9585	42.14	<0.0001
HDL <40 mg/dL (men), HDL <50 mg/dL (women)	Yes	62 951	45.58	7820	40.09	9645	43.49	12 887	46.54	10 707	46.71	11 087	48.02	10 805	47.50	<0.0001
LDL, mg/dL	Median	91 626	100.00	12 057	103.00	14 142	101.00	17 950	100.00	15 332	100.00	16 142	98.00	16 003	98.00	<0.0001
	25^th^		76.00		81.00		78.00		77.00		76.00		74.00		73.00	
	75^th^		128.00		131.00		128.00		128.00		128.00		125.00		125.00	
	Mean		104.37		108.07		105.64		104.87		104.37		102.42		101.87	
	SD		39.89		39.25		39.48		39.48		39.71		40.70		40.23	
	Minimum		30.00		30.00		30.00		30.00		30.00		30.00		30.00	
	Maximum		500.00		483.00		486.00		451.00		444.00		500.00		500.00	
LDL >100 mg/dL	Yes	45 290	32.79	6432	32.98	7215	32.53	8929	32.25	7563	33.00	7622	33.02	7529	33.10	<0.0001
Triglycerides, mg/dL	Median	92 852	122.00	12 499	128.00	14 635	126.00	18 535	124.00	15 569	120.00	15 983	119.00	15 631	119.00	<0.0001
	25^th^		84.00		88.00		87.00		85.00		83.00		81.00		83.00	
	75^th^		181.00		189.00		186.00		185.00		176.00		176.00		177.00	
	Mean		152.79		157.78		156.25		155.57		149.45		148.45		150.01	
	SD		120.74		121.23		120.24		122.71		119.41		119.30		120.92	
	Minimum		5.00		5.00		5.70		6.60		7.00		5.00		5.00	
	Maximum		1998.0		1977.0		1881.0		1813.0		1938.0		1998.0		1935.0	
Triglycerides >150 mg/dL	Yes	33 229	24.06	4762	24.42	5540	24.98	6830	24.67	5319	23.21	5429	23.52	5349	23.52	<0.0001

Categorical data in columns are displayed as count|percent of overall. SD indicates standard deviation; UTD, unable to determine; COPD, chronic obstructive pulmonary disease; CVA, cerebrovascular accident; MI, myocardial infarction; TIA, transient ischemic attack; PCI, percutaneous coronary intervention; CABG, coronary artery bypass graft; LDL, low-density lipoprotein; HDL, high-density lipoprotein; BMI, body mass index; and AMI, acute myocardial infarction.

The distribution of racial/ethnic groups within the overall sample exhibited a significant change over time (Table 1), with a decreasing proportion of Hispanics within the sample, an increasing proportion of African Americans, and a relatively constant proportion of white patients. There was also a significant change in the distribution of insurance status over time (Table 1), with an initial increase in the proportion of Medicaid and uninsured patients and a decrease in the proportion of Medicare patients.

### Clinical Characteristics and Risk Factors of Overall AMI Patient Population Over Time: Univariate Analysis

**Figure 2.**
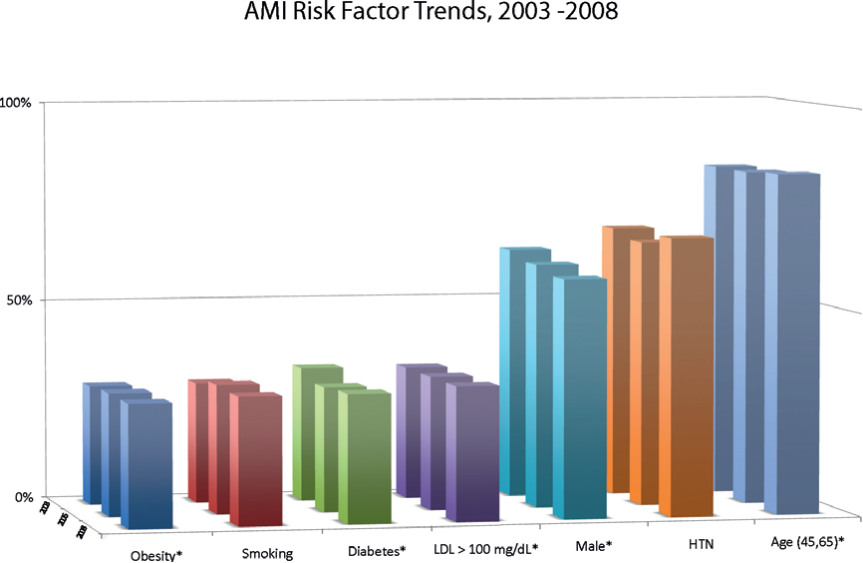
Trends in prevalence of cardiovascular risk factors (age, sex, hypertension, diabetes mellitus, hyperlipidemia, obesity, smoking) from 2003 to 2008 in the overall GWTG-CAD AMI sample. *P<0.05 for trend. HTN indicates hypertension.

Ninety-three percent of the sample reported at least 1 risk factor among 5 modifiable “classic” risk factors (hypertension, hyperlipidemia, current smoking, diabetes mellitus, and obesity), and 69% reported ≥2 such risk factors. The overall prevalence of a history of hypertension remained high at 67.1% and varied little over time (2003, 67.6%; 2008, 68.5%; *P* for trend=0.19). The prevalence of current or recent smoking did not change significantly (2003, 29.9%; 2008, 31.5%; *P* for trend=0.74). The prevalence of a history of hyperlipidemia increased (2003, 32.7%; 2008, 50.4%; *P* for trend <0.0001). The prevalence of a BMI ≥30 kg/m^2^ increased slightly, from 29.6% in 2003 to 30.3% in 2008 (*P* for trend <0.0001). The overall prevalence of diabetes mellitus was 32.0% and decreased over time from 33.2% to 31.7% (*P* for trend <0.0001). The prevalence of total cholesterol >200 mg/dL decreased from 18.5% in 2003 to 15.2% in 2008 (*P* for trend <0.0001), and the prevalence of LDL >100 mg/dL initially trended down from 2003 to 2006 and then seemed to stabilize from 2006 to 2008 (*P* for trend <0.0001). However, the prevalence of “low” HDL (men, <40 mg/dL; women, <50 mg/dL) increased from 40.1% in 2003 to 47.5% in 2008 (*P* for trend <0.0001). There was a significant increase in the proportion of patients with NSTEMI over time (Table 1). Overall trends in the prevalence of key risk factors are depicted in Figure 2.

There were notable differences between patients with NSTEMI and patients with STEMI (Table 2). In general, patients with NSTEMI were significantly more likely to be older and female and to have a greater burden of clinical risk factors—for example, hypertension, diabetes mellitus, and prior myocardial infarction. Conversely, patients with STEMI were more likely to be younger, male, and active smokers and to have a higher prevalence of biochemical risk factors—for example, “low” HDL, “high” LDL, and triglycerides >150 mg/dL.

**Table 2.** STEMI and NSTEMI Patient Profile, 2003–2008

	STEMI (n=45 172)	NSTEMI (n=92 950)	STEMI vs NSTEMI (*P*)
Age, y			
Median	62	69	<0.0001
IQR	22	23	
Mean	62.86	68.16	
SD	14.15	14.2	
Age ≤45 y, %	10.71	6.25	<0.0001
Age ≥65 y, %	43.33	60.14	<0.0001
Male, %	66.53	58.33	<0.0001
Race, %			
Hispanic	7.15	7.34	<0.0001
Black or African American	6.51	7.79	
White	75.55	74.36	
Asian	2.87	3.32	
Insurance status, %			
None/UTD	12.86	6.95	<0.0001
Medicare	24.47	33.30	
Medicaid	5.66	7.22	
Other	48.41	44.49	
Missing	8.60	8.03	
Diabetes mellitus, %	25.56	35.12	<0.0001
Hypertension, %	60.60	70.21	<0.0001
Hyperlipidemia, %	44.44	47.85	<0.0001
Prior MI/CAD, %	23.24	32.60	<0.0001
Current/recent smoking, %	39.82	26.92	<0.0001
BMI ≥30 kg/m^2^, %	30.17	30.37	0.0874
HDL <40 mg/dL (men), <50 mg/dL (women), %	49.35	43.74	0.0283
LDL >100 mg/dL, %	37.92	30.30	<0.0001
Triglycerides >150 mg/dL, %	26.31	22.96	<0.0001

SD indicates standard deviation; STEMI, ST-segment elevation myocardial infarction; NSTEMI, non-ST-segment elevation myocardial infarction; UTD, unable to determine; MI, myocardial infarction; IRQ, interquartile range; CAD, coronary artery disease; BMI, body mass index; HDL, high-density lipoprotein; and LDL, low-density lipoprotein.

### Demographic, Clinical, and Biochemical Characteristics of Patients With STEMI Over Time: Univariate Analysis

As seen in Table 3, in patients with STEMI, the median/IQR ages in 2003 and 2008 were, respectively, 63/22 years and 61/20 years (*P* for trend <0.0001), and the proportion of patients ≥65 years of age decreased (*P*<0.0001). The proportion of patients between 45 and 65 years of age remained stable over time. The sex ratio (number of males/100 females) increased over time (*P*=0.0002). There was a slight but significant decrease in the proportion of non-Hispanic whites (*P*<0.0001) and a decrease in the proportion of Hispanic patients over time (2003, 7.1%; 2008, 6.25%; *P*<0.0001). There was a significant decrease in the prevalence of a history of diabetes mellitus (2003, 28.5%; 2008, 22.93%; *P*<0.0001) and history of hypertension (2003, 63.06%; 2008, 60.46%; *P*<0.0001), although the prevalence of a history of hyperlipidemia increased (2003, 32.36%; 2008, 46.27%; *P*<0.0001). The prevalence of smoking increased (2003, 37.22%; 2008, 41.76%; *P*=0.0002). The prevalence of obesity increased (2003, 29.7%; 2008, 30.82%; *P*<0.0001). The prevalence of “low” HDL (<40 mg/dL in men, <50 mg/dL in women) increased significantly (2003, 42.86%; 2008, 52.32%; *P*<0.0001), and the prevalence of “high” LDL (LDL >100 mg/dL) increased (2003, 37.2%; 2008, 39.95%; *P*<0.0001), as did the prevalence of triglycerides >150 mg/dL (2003, 26.27%; 2008, 27.02%; *P*=0.0004).

**Table 3.** Trends in Demographic, Medical Historical, and Laboratory Characteristics in Patients With STEMI

Variables	Level	Overall (n=45 172)		2003 (n=6706)		2004 (n=7486)		2005 (n=8638)		2006 (n=7640)		2007 (n=7648)		2008 (n=7054)		*P*
Demographics																
Age	Median	45 172	62.00	6706	63.00	7486	62.00	8638	62.00	7640	62.00	7648	60.00	7054	61.00	<0.0001
	25th		52.00		53.00		53.00		53.00		52.00		52.00		52.00	
	75th		74.00		75.00		75.00		75.00		74.00		73.00		72.00	
	Mean		62.86		63.72		63.37		63.15		62.77		62.13		62.06	
	SD		14.15		14.02		14.23		14.32		14.17		14.12		13.94	
	Minimum		18.00		19.00		18.00		18.00		19.00		18.00		20.00	
	Maximum		107.00		102.00		103.00		102.00		104.00		106.00		107.00	
Age ≤45 y	Yes	4837	10.71	666	9.93	775	10.35	930	10.77	826	10.81	866	11.32	774	10.97	0.0079
Age ≥65 y	Yes	19 574	43.33	3132	46.70	3384	45.20	3824	44.27	3281	42.95	3103	40.57	2850	40.40	<0.0001
Age >45, <65 y	Yes	20 761	81.10	2908	81.37	3327	81.11	3884	80.68	3533	81.05	3679	80.95	3430	81.59	0.7926
Sex	Female	14 312	31.68	2220	33.10	2488	33.24	2785	32.24	2390	31.28	2335	30.53	2094	29.69	0.0002
Race	Other	1567	3.47	201	3.00	218	2.91	301	3.48	310	4.06	290	3.79	247	3.50	<0.0001
	Hispanic	3232	7.15	477	7.11	698	9.32	671	7.77	474	6.20	471	6.16	441	6.25	
	Black or African American	2941	6.51	439	6.55	431	5.76	535	6.19	535	7.00	485	6.34	516	7.31	
	White	34 128	75.55	5124	76.41	5499	73.46	6531	75.61	5689	74.46	5931	77.55	5354	75.90	
	Missing	3304	7.31	465	6.93	640	8.55	600	6.95	632	8.27	471	6.16	496	7.03	
Non-Hispanic white	Yes	34 128	75.55	5124	76.41	5499	73.46	6531	75.61	5689	74.46	5931	77.55	5354	75.90	<0.0001
Hispanic	Yes	3232	7.15	477	7.11	698	9.32	671	7.77	474	6.20	471	6.16	441	6.25	<0.0001
Insurance	No insurance/not documented/UTD	5808	12.86	691	10.30	1155	15.43	1309	15.15	1011	13.23	812	10.62	830	11.77	<0.0001
	Medicare	11 053	24.47	1847	27.54	2051	27.40	2201	25.48	1756	22.98	1682	21.99	1516	21.49	
	Medicaid	2555	5.66	300	4.47	443	5.92	559	6.47	438	5.73	433	5.66	382	5.42	
	Other	21 869	48.41	2872	42.83	3702	49.45	4510	52.21	3770	49.35	3733	48.81	3282	46.53	
	Missing	3887	8.60	996	14.85	135	1.80	59	0.68	665	8.70	988	12.92	1044	14.80	
Medical history																
Laboratories																
BMI, kg/m^2^	Median	40 478	27.68	6221	27.46	6963	27.42	8068	27.63	6643	27.64	6571	27.97	6012	28.05	<0.0001
	25th		24.48		24.46		24.32		24.41		24.45		24.58		24.82	
	75th		31.56		31.24		31.11		31.47		31.67		31.88		32.03	
	Mean		28.52		28.31		28.19		28.48		28.50		28.83		28.87	
	SD		6.21		6.11		6.08		6.25		6.09		6.44		6.24	
	Minimum		13.02		13.06		13.17		13.02		13.07		13.19		13.04	
	Maximum		95.20		74.73		76.13		85.41		69.27		95.20		72.66	
BMI ≥30 kg/m^2^	Yes	13 628	30.17	1992	29.70	2182	29.15	2652	30.70	2274	29.76	2354	30.78	2174	30.82	<0.0001
Total cholesterol, mg/dL	Median	33 076	170.00	4737	175.00	5323	172.00	6334	169.00	5581	169.00	5775	167.00	5326	167.00	<0.0001
	25th		142.00		148.00		144.00		141.00		141.00		139.00		139.00	
	75th		201.00		206.00		203.00		200.00		200.00		197.00		199.00	
	Mean		173.71		179.60		176.02		172.51		172.89		170.93		171.45	
	SD		46.70		47.35		46.72		45.21		47.09		46.59		47.02	
	Minimum		10.00		19.00		22.00		10.00		12.00		26.00		50.00	
	Maximum		776.00		776.00		720.00		574.00		667.00		709.00		608.00	
Total cholesterol >200 mg/dL	Yes	8305	18.39	1364	20.34	1424	19.02	1547	17.91	1377	18.02	1322	17.29	1271	18.02	<0.0001
HDL, mg/dL	Median	32 593	36.00	4614	39.00	5218	37.00	6248	35.85	5516	36.00	5719	36.00	5278	36.00	<0.0001
	25th		30.00		32.00		31.00		29.00		29.00		30.00		30.00	
	75th		44.00		47.00		45.00		44.00		44.00		44.00		43.00	
	Mean		38.24		40.61		39.09		37.34		37.69		37.74		37.49	
	SD		12.22		12.48		12.63		12.83		12.16		11.28		11.57	
	Minimum		0.00		0.00		0.00		0.00		5.00		5.00		3.00	
	Maximum		100.00		100.00		98.00		100.00		99.00		99.00		100.00	
HDL <40 mg/dL (men), HDL <50 mg/dL (women)	Yes	22 292	49.35	2874	42.86	3471	46.37	4414	51.10	3829	50.12	4013	52.47	3691	52.32	<0.0001
LDL, mg/dL	Median	32 264	103.00	4471	106.00	5074	104.00	6087	103.00	5450	104.00	5754	102.00	5428	102.00	<0.0001
	25th		80.00		82.00		80.00		80.00		80.00		78.00		78.00	
	75th		130.00		132.00		130.00		130.00		131.00		128.00		129.00	
	Mean		107.23		109.61		107.76		107.04		107.44		105.82		106.27	
	SD		39.45		38.65		39.50		38.68		38.76		39.83		41.08	
	Minimum		30.00		30.00		30.00		30.00		30.00		30.00		30.00	
	Maximum		500.00		400.00		486.00		451.00		399.00		500.00		500.00	
LDL >100 mg/dL	Yes	17 130	37.92	2497	37.24	2725	36.40	3207	37.13	2905	38.02	2978	38.94	2818	39.95	<0.0001
Triglycerides, mg/dL	Median	32 641	124.00	4636	127.00	5221	127.00	6269	124.00	5526	123.00	5701	122.00	5288	124.00	<0.0001
	25th		86.00		90.00		88.00		85.00		84.00		83.00		85.00	
	75th		182.00		189.00		187.00		181.00		179.00		178.00		181.00	
	Mean		153.62		159.80		157.39		152.10		151.82		148.98		153.17	
	SD		119.97		128.63		122.33		115.21		118.84		117.06		119.28	
	Minimum		5.00		5.00		5.70		8.00		13.00		12.00		5.00	
	Maximum		1998.0		1977.0		1539.0		1813.0		1659.0		1998.0		1863.0	
Triglycerides >150 mg/dL	Yes	11 886	26.31	1762	26.27	1985	26.52	2275	26.34	1971	25.80	1987	25.98	1906	27.02	0.0004

Categorical data in columns are displayed as count|percent of overall. SD indicates standard deviation; UTD, unable to determine; STEMI, ST-segment elevation myocardial infarction; BMI, body mass index; HDL, high-density lipoprotein; and LDL, low-density lipoprotein.

### Demographic, Clinical, and Biochemical Characteristics of Patients With NSTEMI Over Time: Univariate Analysis

As seen in Table 4, in patients with NSTEMI, the median/IQR ages in 2003 and 2008 were, respectively, 70/21 years and 69/22 years (*P* for trend=0.004). The proportion of patients between 45 and 65 years of age increased slightly. In contrast to the patients with STEMI, sex ratio decreased over time (*P*<0.0001). There was a trend toward an increase in the proportion of non-Hispanic whites, and the proportion of Hispanic patients decreased over time (2003, 8.38%; 2008, 6.18%; *P*<0.0001). Consistent with the older age of patients with NSTEMI, there was a higher proportion of Medicare-insured patients. The prevalence of a history of diabetes mellitus marginally decreased over time (2003, 35.62%; 2008, 35.55%; *P*=0.0327). The prevalence of a history of hypertension increased further over time (2003, 69.92%; 2008, 72.10%; *P*=0.0162), and the prevalence of a history of hyperlipidemia increased from 32.8% in 2003 to 52.21% in 2008 (*P*<0.0001). There was a marginal increase in the prevalence of smoking (2003, 26.08%; 2008, 26.91%; *P*=0.0224). The prevalence of obesity increased marginally, from 29.57% in 2003 to 29.99% in 2008 (*P*<0.0001). The prevalence of “low” HDL increased from 38.65% in 2003 to 45.34% in 2008 (*P*<0.0001), whereas the prevalence of “high” LDL decreased marginally (2003, 30.75%; 2008, 30.02%; *P*<0.0001).

**Table 4.** Trends in Demographic, Medical Historical, and Laboratory Characteristics in Patients With NSTEMI

Variables	Level	Overall (n=92 950)		2003 (n=12 798)		2004 (n=14 691)		2005 (n=19 051)		2006 (n=15 281)		2007 (n=15 438)		2008 (n=15 691)		*P*
Demographics																
Age	Median	92 950	69.00	12 798	70.00	14 691	69.00	19 051	69.00	15 281	69.00	15 438	69.00	15 691	69.00	0.0038
	25th		57.00		58.00		57.00		57.00		57.00		58.00		58.00	
	75th		80.00		79.00		79.00		80.00		79.00		80.00		80.00	
	Mean		68.16		68.20		68.02		68.05		67.74		68.35		68.64	
	SD		14.24		13.90		14.20		14.34		14.26		14.29		14.33	
	Minimum		18.00		19.00		19.00		19.00		20.00		18.00		19.00	
	Maximum		106.00		104.00		106.00		104.00		105.00		106.00		105.00	
Age ≤45 y	Yes	5808	6.25	780	6.09	942	6.41	1266	6.65	986	6.45	942	6.10	892	5.68	0.0291
Age ≥65 y	Yes	55 889	60.14	7848	61.32	8870	60.38	11 436	60.03	8957	58.62	9259	59.98	9529	60.73	0.1567
Age >45, <65 y	Yes	31 243	84.32	4170	84.24	4879	83.82	6349	83.37	5338	84.41	5237	84.75	5270	85.52	0.0050
Sex	Female	37 184	40.00	5031	39.31	5877	40.00	7604	39.91	5999	39.26	6251	40.49	6422	40.93	<0.0001
Race	Other	3857	4.15	418	3.27	463	3.15	832	4.37	649	4.25	705	4.57	790	5.03	<0.0001
	Hispanic	6819	7.34	1072	8.38	1325	9.02	1498	7.86	1105	7.23	850	5.51	969	6.18	
	Black or African American	7240	7.79	951	7.43	1132	7.71	1357	7.12	1243	8.13	1207	7.82	1350	8.60	
	White	69122	74.36	9543	74.57	10593	72.11	14048	73.74	11196	73.27	11989	77.66	11753	74.90	
	Missing	5912	6.36	814	6.36	1178	8.02	1316	6.91	1088	7.12	687	4.45	829	5.28	
Non-Hispanic White	Yes	69 122	74.36	9543	74.57	10 593	72.11	14 048	73.74	11 196	73.27	11 989	77.66	11 753	74.90	<0.0001
Hispanic	Yes	6819	7.34	1072	8.38	1325	9.02	1498	7.86	1105	7.23	850	5.51	969	6.18	<0.0001
Insurance	No insurance/not documented/UTD	6460	6.95	704	5.50	1206	8.21	1649	8.66	1182	7.74	874	5.66	845	5.39	<0.0001
	Medicare	30 956	33.30	4645	36.29	5106	34.76	6685	35.09	4754	31.11	4695	30.41	5071	32.32	
	Medicaid	6713	7.22	796	6.22	1172	7.98	1380	7.24	1210	7.92	1098	7.11	1057	6.74	
	Other	41 358	44.49	4847	37.87	6815	46.39	9119	47.87	7103	46.48	6982	45.23	6492	41.37	
	Missing	7463	8.03	1806	14.11	392	2.67	218	1.14	1032	6.75	1789	11.59	2226	14.19	
Medical history																
Diabetes mellitus	Yes	30 873	35.12	4255	35.62	5087	36.14	6444	35.23	4968	33.96	5010	34.35	5109	35.55	0.0327
Hyperlipidemia	Yes	42 062	47.85	3925	32.86	6449	45.82	9213	50.36	7538	51.52	7434	50.97	7503	52.21	<0.0001
Hypertension	Yes	61 713	70.21	8353	69.92	9886	70.23	12 824	70.10	10 150	69.37	10 139	69.51	10 361	72.10	0.0162
Prior MI/CAD	Yes	28 658	32.60	2990	25.03	3266	23.20	3954	21.61	5252	35.90	6547	44.89	6649	46.27	<0.0001
CVA/TIA	Yes	8693	9.89	1160	9.71	1311	9.31	1536	8.40	1349	9.22	1669	11.44	1668	11.61	<0.0001
Medical history panel missing	Yes	5047	5.43	852	6.66	615	4.19	758	3.98	650	4.25	852	5.52	1320	8.41	<0.0001
Smoking	Yes	25 022	26.92	3338	26.08	4022	27.38	5108	26.81	4195	27.45	4137	26.80	4222	26.91	0.0224
Laboratories																
BMI, kg/m^2^	Median	82 649	27.53	11 802	27.36	13 725	27.40	17 602	27.45	13 300	27.61	13 154	27.80	13 066	27.81	<0.0001
	25th		24.09		24.09		23.96		24.02		24.12		24.20		24.17	
	75th		31.86		31.45		31.51		31.64		32.10		32.28		32.14	
	Mean		28.54		28.28		28.31		28.41		28.69		28.83		28.76	
	SD		6.81		6.59		6.61		6.70		6.95		7.09		6.92	
	Minimum		13.03		13.05		13.03		13.04		13.08		13.04		13.15	
	Maximum		99.27		99.27		96.95		98.41		88.71		97.00		96.88	
BMI ≥30 kg/m^2^	Yes	28 232	30.37	3784	29.57	4454	30.32	5878	30.85	4669	30.55	4741	30.71	4706	29.99	<0.0001
Total cholesterol, mg/dL	Median	61 018	165.00	8054	172.00	9545	168.00	12 415	166.00	10 173	163.00	10 394	161.00	10 437	160.00	<0.0001
	25th		136.00		143.00		140.00		138.00		134.00		132.00		131.00	
	75th		198.00		205.00		200.00		199.00		196.00		194.00		194.00	
	Mean		169.62		176.54		172.62		170.66		167.81		166.27		165.41	
	SD		48.45		47.60		47.68		48.17		48.86		49.23		48.15	
	Minimum		11.00		15.00		16.00		16.00		11.00		21.00		18.00	
	Maximum		827.00		574.00		624.00		592.00		660.00		827.00		642.00	
Total cholesterol >200 mg/dL	Yes	14 246	15.33	2240	17.50	2345	15.96	2957	15.52	2284	14.95	2226	14.42	2194	13.98	<0.0001
HDL, mg/dL	Median	60 008	37.00	7816	39.00	9356	38.00	12 242	36.00	10 022	36.00	10 271	37.00	10 301	37.00	<0.0001
	25th		30.00		32.00		31.00		29.00		30.00		30.00		30.00	
	75th		46.00		47.00		47.00		45.00		45.00		45.00		45.00	
	Mean		39.05		40.55		39.73		38.23		38.62		38.90		38.86	
	SD		13.24		12.88		13.49		13.92		13.23		12.72		12.81	
	Minimum		0.00		0.00		0.00		0.00		0.00		0.00		2.00	
	Maximum		100.00		100.00		100.00		100.00		100.00		100.00		100.00	
HDL <40 mg/dL	Yes	35 016	37.67	4112	32.13	5221	35.54	7339	38.52	6044	39.55	6098	39.50	6202	39.53	<0.0001
HDL <40 mg/dL (men), HDL <50 mg/dL (women)	Yes	40 659	43.74	4946	38.65	6174	42.03	8473	44.48	6878	45.01	7074	45.82	7114	45.34	<0.0001
LDL, mg/dL	Median	59 362	98.00	7586	102.00	9068	100.00	11 863	99.00	9882	97.00	10 388	95.00	10 575	95.00	<0.0001
	25th		74.00		80.00		77.00		75.00		74.00		71.00		70.00	
	75th		126.00		130.00		127.00		127.00		126.00		123.00		123.00	
	Mean		102.82		107.16		104.45		103.76		102.68		100.54		99.60	
	SD		40.04		39.58		39.42		39.84		40.13		41.06		39.60	
	Minimum		30.00		30.00		30.00		30.00		30.00		30.00		30.00	
	Maximum		500.00		483.00		439.00		401.00		444.00		500.00		476.00	
LDL >100 mg/dL	Yes	28 160	30.30	3935	30.75	4490	30.56	5722	30.04	4658	30.48	4644	30.08	4711	30.02	<0.0001
Triglycerides, mg/dL	Median	60 211	121.00	7863	128.00	9414	126.00	12 266	125.00	10 043	119.00	10 282	117.00	10 343	117.00	<0.0001
	25th		84.00		88.00		87.00		85.00		82.00		81.00		81.00	
	75th		181.00		189.00		185.00		186.00		174.00		176.00		174.00	
	Mean		152.33		156.59		155.62		157.34		148.14		148.15		148.39	
	SD		121.15		116.64		119.07		126.35		119.72		120.52		121.72	
	Minimum		5.00		14.00		8.40		6.60		7.00		5.00		14.00	
	Maximum		1940.0		1940.0		1881.0		1750.0		1938.0		1939.0		1935.0	
Triglycerides >150 mg/dL	Yes	21 343	22.96	3000	23.44	3555	24.20	4555	23.91	3348	21.91	3442	22.30	3443	21.94	<0.0001

Categorical data in columns are displayed as count|percent of overall. SD indicates standard deviation; UTD, unable to determine; MI, myocardial infarction; CVA, cerebrovascular accident; TIA, transient ischemic attack; NSTEMI, non-ST-segment elevation myocardial infarction; CAD, coronary artery disease; BMI, body mass index; HDL, high-density lipoprotein; and LDL, low-density lipoprotein.

### Sensitivity Analysis

Sensitivity analysis on the core data set (cf. Methods) indicated excellent quantitative and qualitative agreement with the overall data set findings. Specifically, the frequency of missing medical history data was 6.35% in the core data set and 5.91% in the overall data set. Median age (67 years) and mean age (66.3 years) in the core data set were identical to the overall data set, and the trends over time were directionally similar. Sex ratios were numerically similar in the core and overall data sets, although the sex ratio trend in the core data set failed to reach statistical significance. Numerically and directionally similar trends in the prevalences of diabetes mellitus, hypertension, and hyperlipidemia were also in close agreement, as were the trends in obesity prevalence and “low” HDL.

### Trends in Clinical Characteristics and Risk Factors of STEMI Patient Population: Multivariable Analysis

After adjustment for multiple potential confounding variables, including other risk factors (Table 5), the increase over time in the proportion of patients between 45 and 65 years of age was significant, along with increases in the prevalences of obesity and “low” HDL. However, there were significant decreases over time in the prevalences of hypertension, diabetes mellitus, prior AMI, and current/recent smoking, as well as decreases in the prevalences of “high” LDL and triglycerides >150 mg/dL.

**Table 5.** ORs for Outcomes for Calendar Quarter (Per 6-Year Increase) With Adjustment for Potential Confounders*: STEMI Group

Outcome	Total N (45 172)	Unadjusted OR	Lower (95% CI for Unadjusted OR)	Upper (95% CI for Unadjusted OR)	Unadjusted *P*	Adjusted OR	Lower (95% CI for Adjusted OR)	Upper (95% CI for Adjusred OR)	Adjusted *P*
Demographics									
Age ≤45 y	45 172	1.100	0.970	1.248	0.136	0.873	0.759	1.005	0.059
Age ≥65 y	45 172	0.756	0.685	0.835	<0.001	1.053	0.879	1.261	0.579
Age >45, <65 y	25 598	1.041	0.921	1.176	0.521	1.148	1.009	1.306	0.036
Sex, male	44 364	1.166	1.063	1.278	0.001	1.071	0.976	1.175	0.149
White race	44 077	1.021	0.796	1.310	0.869	1.010	0.780	1.307	0.942
Hispanic ethnicity	44 077	1.174	0.933	1.476	0.171	1.113	0.888	1.395	0.354
Medical history									
Diabetes mellitus	42 051	0.671	0.595	0.757	<0.001	0.718	0.643	0.802	<0.001
Hypertension	42 051	0.800	0.700	0.915	0.001	0.813	0.704	0.939	0.005
Prior MI	42 051	0.681	0.589	0.787	<0.001	0.735	0.631	0.856	<0.001
Current or recent smoking	44 112	0.984	0.898	1.078	0.727	0.890	0.802	0.987	0.028
Laboratories									
BMI ≥30 kg/m^2^	40 478	1.264	1.146	1.394	<0.001	1.232	1.119	1.357	<0.001
LDL >100 mg/dL	32 264	0.789	0.708	0.879	<0.001	0.688	0.605	0.781	<0.001
HDL <40 mg/dL (men), <50 mg/dL (women)	32 593	1.588	1.357	1.859	<0.001	1.667	1.396	1.992	<0.001
TG >150 mg/dL	32 641	0.866	0.767	0.978	0.020	0.821	0.731	0.922	<0.001

COPD indicates chronic obstructive pulmonary disease; CI, confidence interval; MI, myocardial infarction; TIA, transient ischemic attack; TG, triglycerides; STEMI, ST-segment elevation myocardial infarction; BMI, body mass index; LDL, low-density lipoprotein; and HDL, high-density lipoprotein.

* Variables in the model: age, sex, race (white, black, Hispanic, other), BMI, insurance, atrial fibrillation, COPD/asthma, cerebrovascular accident/TIA, diabetes mellitus, hyperlipidemia, hypertension, peripheral vascular disease, prior MI, heart failure, dialysis, renal insufficiency, smoking, geographic region, number of beds, teaching status, and cardiac surgery on site.

### Trends in Clinical Characteristics and Risk Factors of NSTEMI Patient Population: Multivariable Analysis

After adjustment for multiple confounding variables, including other risk factors (Table 6), there were significant increases over time in the proportion of patients between 45 and 65 years of age, whereas the proportion of “younger” patients (≤45 years) decreased. The proportion of women increased over time, as did the proportion of Hispanic patients. The prevalence of diabetes mellitus decreased over time, whereas the prevalence of obesity increased. The prevalence of “low” HDL increased significantly, whereas the prevalence of “high” LDL decreased over time.

**Table 6.** ORs for Outcomes for Calendar Quarter (Per 6-Year Increase) With Adjustment for Potential Confounders*: NSTEMI Group

Outcome	Total N (92 950)	Unadjusted OR	Lower (95% CI for Unadjusted OR)	Upper (95% CI for Unadjusted OR)	Unadjusted *P*	Adjusted OR	Lower (95% CI for Adjusted OR)	Upper (95% CI for Adjusted OR)	Adjusted *P*
Demographics									
Age ≤45 y	92 950	0.884	0.776	1.008	0.065	0.804	0.689	0.939	0.006
Age ≥65 y	92 950	1.014	0.943	1.090	0.715	1.168	0.975	1.400	0.093
Age >45, <65 y	37 051	1.157	1.024	1.308	0.019	1.213	1.054	1.396	0.007
Sex, male	91 401	0.896	0.833	0.964	0.003	0.921	0.850	0.997	0.043
White race	90 866	1.264	0.850	1.879	0.247	1.059	0.730	1.535	0.762
Hispanic ethnicity	90 866	1.211	1.065	1.377	0.003	1.232	1.101	1.379	<0.001
Medical history									
Diabetes mellitus	87 903	0.920	0.852	0.994	0.034	0.888	0.814	0.968	0.007
Hypertension	87 903	1.003	0.884	1.138	0.965	0.886	0.779	1.007	0.065
Prior MI	87 903	0.703	0.589	0.840	<0.001	0.706	0.591	0.844	<0.001
Current or recent smoking	90 591	0.867	0.798	0.943	<0.001	0.944	0.858	1.038	0.232
Laboratories									
BMI ≥30 kg/m^2^	82 649	1.192	1.112	1.278	<0.001	1.233	1.147	1.325	<0.001
LDL >100 mg/dL	59 362	0.726	0.669	0.787	<0.001	0.665	0.604	0.732	<0.001
HDL <40 mg/dL (men), <50 mg/dL (women)	60 008	1.437	1.232	1.677	<0.001	1.657	1.388	1.978	<0.001
TG >150 mg/dL	60 211	0.736	0.675	0.801	<0.001	0.733	0.665	0.807	<0.001

COPD indicates chronic obstructive pulmonary disease; CI, confidence interval; MI, myocardial infarction; TIA, transient ischemic attack; TG, triglycerides; NSTEMI, non-ST-segment elevation myocardial infarction; BMI, body mass index; LDL, low-density lipoprotein; and HDL, high-density lipoprotein.

* Variables in the model: age, sex, race (white, black, Hispanic, other), BMI, insurance, atrial fibrillation, COPD/asthma, cerebrovascular accident / TIA, diabetes mellitus, hyperlipidemia, hypertension, peripheral vascular disease, prior MI, heart failure, dialysis, renal insufficiency, smoking, geographic region, number of beds, teaching status, and cardiac surgery on site.

### Sensitivity Analysis

In general, there was quantitative and qualitative agreement between the core data sets and the overall stratum-specific analyses. In the STEMI group, the analyses differed only in the magnitude of the coefficient for the decrease in hypertension prevalence, whereas in the NSTEMI group, the analyses differed only in the magnitude of the coefficients for the changes in sex ratio and hypertension prevalence.

## Discussion

The present analysis of the clinical, demographic, and biochemical characteristics of patients with AMI admitted to hospitals participating in the AHA GWTG-CAD quality-improvement initiative from 2003 to 2008 suggests that the cumulative risk factor burden in patients with AMI remained substantial. Favorable decreases in the prevalences of several “classic” risk factors over this interval were offset by increases in the prevalences of obesity and “low” HDL and suggest that metabolic derangements are likely to remain important contributors to overall risk factor burden.

The present observations are in agreement with previous reports from dedicated registries of patients with AMI^12–13^ and population-based studies,^14–15^ which described an increase in the NSTEMI/STEMI ratio over time. Although some of this increase has been attributed to a change in the diagnostic criteria for AMI around 2000,^16^ not all of the increase in the proportion of NSTEMI can be attributed to this transition.^15,17–18^ All patients in the present analysis were enrolled from 2003 forward and thus were ascertained with standardized post-transition criteria.

Our data are also in agreement with prior studies reporting the risk factor burden in patients with AMI.^1–3^ Despite high prevalence of a history of hyperlipidemia and hypertension, the recorded numerical values for admission blood pressure (data not shown), LDL, and total cholesterol in the GWTG-CAD registry are consistent with the increasing extent of antihypertensive and lipid-lowering treatment in the general US population.^19–20^

The present data mirror previously reported magnitudes and trends in the prevalence of obesity in AMI registries^12–13^ and population-based studies.^14,19,21^ However, the increase in the prevalence of obesity in the general population might not be continuing at the same rate in more recent years.^22–23^ The small numerical, albeit statistically significant, increase in the prevalence of BMI ≥30 kg/m^2^ in the present sample of patients with AMI is in agreement with these latter reports. The clinical relevance of an overall prevalence of obesity of 30% in this sample of patients with AMI should not be overlooked, given the strong associations among obesity, diabetes mellitus, hypertension, and dyslipidemia. The observed downward trend in the unadjusted and adjusted prevalences of diabetes mellitus in our study remains unexplained and is at odds with prior observations in patients with AMI,^12–13^ although it is numerically consistent with a more recent nested cohort population-based study.^15^ The present data should be viewed in the broader clinical context of, on average, a prevalence of diabetes mellitus of 30% in patients with AMI,^6,14,21^ depending on the diagnostic criteria used. The overall prevalence of diabetes mellitus was higher in patients with NSTEMI, whereas the magnitude of change in the prevalence of diabetes mellitus in patients with NSTEMI was less than that observed in patients with STEMI, which underscores the importance of stratum-specific analysis.

The additional information presented herein about a significant trend for the increase in prevalence of “low” HDL is in agreement with previous reports of an increase in prevalence of metabolic derangements in patients with AMI^24^ as well as in the adult US population.^25^

### Implications of Changes in Demographic Composition of the Current AMI Sample

The changes in the age and sex distributions of the GWTG-CAD AMI population from 2003 to 2008, as shown in Figure 1A and 1B, parallel the changes seen in the US population in the first decade of this century,^5^ with the fastest rate of growth noted in the 45- to 64-year age group.^5^ This group comprises the initial cohorts of the “Baby Boom” generation as they enter the age range in which the risk of AMI begins to increase steeply.^6^ The increased prevalence of poor cardiovascular health behaviors and health factors in middle (40 to 64 years) and older (≥65 years) age groups in the US population over the identical time period as the present study provides additional insight into the correspondence between specific characteristics in patients with AMI and adults of similar age in the general population.^26^ In a separate report from the National Health and Nutrition Examination Survey encompassing the years 1988–2010, the prevalence of smoking decreased, and the prevalences of desirable levels of untreated blood pressure and total cholesterol were unchanged, whereas the prevalences of desirable levels of BMI and fasting glucose decreased,^27^ indicative of a persistently elevated risk factor burden in the general US population.

The public health implications and relevance of these observations and correlations are clear.^28–30^ The prevalence of risk factors, and their trends over time, in patients with AMI point to additional need for risk factor intervention at the population level.^31–32^ The present data from 2003 to 2008, however, only begin to suggest a population momentum effect resulting from the age cohorts comprising the “Baby Boom” generation. Even static levels of age-specific prevalences, when multiplied by the increasing number of subjects at risk due to population momentum, will result in an increase in the overall risk factor burden.

### Limitations

The limitations of the present analysis relate chiefly to the use of registry data. It is recognized that there are many potential sources of selection bias in any registry and that the patients in the AHA GWTG-CAD registry might not be representative of all patients with AMI. Similarities to, as well as differences from, the published literature have been noted. Participation in the GWTG-CAD quality-improvement program is voluntary, and as such, the program is likely to include higher-performing hospitals. However, such potential selection bias is unlikely to affect the type, or number, of patient(s) presenting with an AMI, nor are the prevalences of underlying risk factors likely to be affected. Data could be influenced by both drop-in and drop-out of participating hospitals. Sensitivity analysis limited to those hospitals participating in each year revealed substantially similar trends and associations among key risk factors, with few exceptions. Participation in the GWTG-CAD program calls for consecutive enrollment of patients, as is appropriate for performance (per Centers for Medicare and Medicaid Services) and quality-improvement (per Joint Commission) initiatives. Compliance, or the number of patients enrolled per site per year, did not change over time among core sites (*P* for trend=0.17). The analysis of data collected over 6 years from >100 000 patients is likely to be more representative of “real-world” patients with AMI than an analysis from any one region or in any one year would be. The GWTG-CAD program includes sites from all regions of the United States and includes academically affiliated as well as community-based hospitals. Patients in the GWTG-Stroke performance-improvement program, a group not substantially dissimilar from patients with AMI with regard to cardiovascular risk factors, have been shown to be similar to patients in non–GWTG-participating centers.^33^ However, at the present time, there are no comparable studies in patients with CAD/AMI. Changes in professional and societal awareness of the presence and importance of sex-specific differences in cardiovascular disease at the time of this study^34^ could have had an important, albeit unquantifiable, effect on our findings. However, a recent study failed to identify differences in the time to hospital presentation among women with AMI after a national awareness campaign.^35^

Data were collected by chart review and are dependent on the accuracy and completion of documentation and abstraction. All data are entered at the site by highly trained individuals with experience in data entry. The GWTG database features carefully defined data entries, standardized diagnostic criteria throughout, and regular quality assessment. Importantly, the GWTG database includes only patients with confirmed AMI diagnosis at discharge and avoids many of the sources of information bias when the diagnosis is based on admission characteristics. These data and inferences from the data could, however, be limited by potential bias resulting from the inability of disadvantaged and minority groups to access medical care. Such patients are not, by definition, included in the GWTG-CAD data set and cannot be evaluated. The inferences with regard to changes in the prevalence of risk factors suggested by these data apply to the overall patient population.

The magnitudes of the reported main outcome measures of association—the OR for a change in prevalence of a given risk factor per 1 year—were small and initially suggested little clinically significant change from year to year, despite their statistical significance. We chose to report the cumulative OR for the change in prevalence of characteristics over the 6-year observation period in an effort to underscore their clinical significance. Statistical methodology dictates that the (adjusted) ORs must be interpreted in the context of all other covariates being fixed. From clinical and epidemiological perspectives, multiple covariates are not infrequently identified in the same individual—for example, diabetes mellitus, hypertension, and obesity. Statistical attempts to “isolate” changes in one of several highly associated variables might result in unstable or misleading estimates of a true association.

It is acknowledged that the use of an OR as an approximation of relative risk, or risk ratio, is problematic when prevalence is high. The majority of the characteristics and risk factors reported here have a high prevalence, and calculation of prevalence ratios and their changes would be more appropriate.^36^ However, qualitative inferences from this study remain valid.

In conclusion, the present analysis, based on >100 000 patients with AMI from 2003 to 2008, indicates that there were clinically and statistically significant changes over time in the risk factors and characteristics assessed. Increases in the prevalence of women, NSTEMI, and patients 45 to 65 years of age, when viewed from an epidemiological perspective, have important implications for the identification of further opportunities for risk factor modification. Continued increases in the prevalence of obesity and low HDL over the next decade, along with persistently high prevalences of hypertension and diabetes mellitus, particularly in the growing segment of patients with NSTEMI, could offset the beneficial clinical effects of decreasing trends in other risk factors and could result in higher disease burden and post-AMI morbidity in AMI survivors.^37^
